# Monitoring and stopping Hymenoptera venom immunotherapy: Contribution of IgE blocking activity

**DOI:** 10.1016/j.jacig.2024.100329

**Published:** 2024-08-24

**Authors:** Julie Poulat, Elisabeth Bellet-Fraysse, François Touraine, Camille Coumes-Salomon, Boris Melloni, François Belle-Moudourou, Stéphane Charret, François Vincent, Ahmed Boumediene

**Affiliations:** a Department of Pneumology and Allergology, Dupuytren Hospital, Centre Hospitalier Universitaire de Limoges, Limoges, France; b Department of Immunology, Centre Hospitalier Universitaire de Limoges, Limoges, France; c Department of Experimental Pneumology, INSERM U1308, University of Medicine, Limoges, France

**Keywords:** Allergy, Hymenoptera, allergen immunotherapy, IgE blocking activity

## Abstract

**Background:**

Hymenoptera venom allergy is a public health issue and has an undeniable impact on quality of life. Allergen immunotherapy (AIT) has shown long-term efficacy in this severe and potentially lethal allergy. However, no biomarker can predict the effectiveness of this treatment.

**Objectives:**

We evaluated the contribution of IgE blocking activity, a functional biomarker carried out in our center using flow cytometry, to predict the efficacy of AIT.

**Methods:**

This retrospective study from 1985 to 2022 describes in detail the demographic, clinical, and biological characteristics of patients who benefited from AIT with Hymenoptera venom at the University Hospital of Limoges. The outcome measure used was the presence of anaphylactic reaction (grade I to IV according to Ring and Messmer) in case of a new sting after discontinuation of AIT.

**Results:**

Our study, mainly composed of patients allergic to *Vespula* wasp venom, did not emphasize the interest of IgE blocking activity in the prediction of a relapse after a new sting. However, this inhibition showed a significant correlation with the amount of IgG_4_ antibodies.

**Conclusion:**

There is no biomarker that can help make the decision of stopping AIT. However, low levels of IgE blocking activity may suggest a likelihood of relapse. Serum IgG_4_, in correlation with IgE blocking activity, could be useful for monitoring treatment response. Additional studies are necessary to gain a thorough understanding of the composition of inhibitory antibodies.

Insects from the family Hymenoptera are frequently in contact with humans, which leads to public health issues such as allergies. This allergy is the second leading cause of mortality due to anaphylaxis.^1^

In cases of large local reactions or systemic reactions, an allergy assessment is recommended.^2^^,^^3^ It consists in skin tests (prick tests and intradermal reactions) and immunology monitoring (specific IgE antibodies to the implicated venom and its recombinants, serum tryptase level). Basophil activation test (BAT) and IgE blocking activity are performed in specialized flow cytometry facilities.^4^, ^5^, ^6^, ^7^ The only treatment that provides long-term effectiveness is allergen-specific immunotherapy (AIT). AIT is recommended for 5 years in cases of severe initial reactions. In certain cases, such as mast cell disorders, AIT may be lifelong.^8^ AIT has a good efficacy rate—approximately 77-84% with bee venom and 91-96% with wasp venom.^9^, ^10^, ^11^ So far, there is no predictive and efficient biomarker to help decide whether to discontinue AIT.

Concerning immunologic mechanism, at a very early stage, a rapid FcεRI-basophil desensitization with an immediate inhibition of histamine and other basophil mediators release is observed. Another mechanism involves an increase in the proportion of regulatory B and T cells in the peripheral blood. Regulatory T cells possess a suppressive capacity acting at different levels of immune mechanisms (suppression of dendritic cell activation, metabolic disruption mechanisms), and they secrete several cytokines, including IL-10.^12^ The latter is a regulatory cytokine of inflammatory responses and a potent suppressor of many effector cells, such as mast cells and B cells. It also acts by inhibiting the production of IgE and inducing IgG and IgA production.^13^

Regulatory B cells are a heterogeneous group of immunosuppressive B-cell subsets. They suppress effector T-cell responses and induce regulatory T-cell differentiation, inhibit dendritic cell maturation, and promote IgG_4_ production.

Among the mechanisms proposed to account for AIT efficacy are changes in antibody levels and their activity. The successful desensitization to venom during the early stages of AIT may be a result of increased allergen-specific blocking IgA, IgG_1_, and IgG_4_ antibodies.^14^ IgE blocking activity has been described since the 1980s; blocking antibodies can compete with serum IgE (sIgE) and thus prevent IgE–allergen interaction. IgG_4_ antibodies also stop the allergen-induced memory IgE production by blocking low-affinity receptors on B cells, resulting in inhibition of IgE-facilitated presentation of allergens to T cells.

The decrease in specific IgG_4_ and IgE blocking activity, along with the endurance of clinical tolerance after venom removal, indicates the existence of additional mechanisms contributing to long-term tolerance.^12^^,^^15^ Long-term protection obtained by AIT (low relapse after cessation) is probably mediated by numerous immunologic mechanisms that are not well understood.^12^

In our immunology department, we have a history of using IgE blocking activity as a parameter to guide the decision to stop AIT. Considering that AIT increases the production of protective immunoglobulins that inhibit IgE activity, measuring these blocking factors would help demonstrate this activity inhibition. This functional biomarker is used in cases of uncertainty regarding discontinuing AIT, especially when there has been no documented new sting and when the allergy assessment at the end of treatment showed little change compared to the first assessment. A strong positivity of IgE blocking activity, corresponding to an inhibition percentage of 95%, would indicate that discontinuation is appropriate.

## Methods

### Study design and subjects

This study was a retrospective single-center observational study. Patients who benefited from AIT for Hymenoptera venom at the University Hospital of Limoges were selected.

The inclusion criteria were as follows: patients who completed AIT for Hymenoptera venom at the University Hospital of Limoges; and patients who underwent testing for IgE blocking activity at the end or after discontinuation of AIT. The exclusion criteria were AIT duration of less than 5 years and incomplete allergy assessment (skin prick test + IgE before and after AIT).

We collected data related to patients’ professional and medical background, including demographic data (sex, age at first allergy consultation, occupational risk), medical history (hypertension, autoimmune disease, asthma, chronic obstructive pulmonary disease, atopy, heart disease, mastocytosis, diabetes, cancer), medical data (initial reaction after a sting according to Ring and Messmer classification, type of venom used for AIT, start and end dates of AIT, results of skin tests before and after AIT as well as in the years after discontinuation, new stings during or after AIT, any resulting clinical reactions), and biological data (levels of sIgE, sIgG_4_, sIgA, and IgE blocking activity before and after AIT as well as in the years after discontinuation, basal tryptase levels).

### Allergen immunotherapy

AIT is a 2-step procedure consisting of a buildup phase and a maintenance phase. The buildup phase protocol is based on a progressive increase in the doses over a few hours, from 0.1 μg/mL to 100 μg/mL, with an interval of 30 minutes between injections, followed by boosters at 15, 30, and 45 days. Subcutaneous injections in the maintenance phase are usually provided in 4-week intervals in the first 2 years of treatment, every 6 weeks in the second year of treatment, and every 8 weeks from the third to fifth years of AIT.

The pharmaceutical origin of products for AIT and skin tests was Jubilant Pharma, distributed by Stallergenes Greer.

### Skin tests

Skin intradermal tests (IDTs) were performed by progressively increasing the dilution by a tenth, starting at 0.001 μg/mL (10^−5^) and ending at 10 μg/mL (10^−1^). A positive control was performed with histamine. The skin IDT was read 20 minutes after administration, and positivity was determined when the wheal size was at least 3 mm larger than the initial wheal.

### Quantification of allergen-specific IgE, IgG_4_, and IgA antibodies and tryptase

Specific IgE and IgG_4_ measurements were performed retrospectively using the ImmunoCAP technique on a Phadia 250 instrument and IgA with Phadia 100 instrument. When the serum samples from the included patients were stored at the Limoges serum bank, IgG_4_ and IgA were measured at the initiation of AIT, at the end of AIT, and during follow-up after discontinuation.

In addition, we conducted a study analyzing 30 samples of healthy individuals to establish the baseline levels of specific IgG_4_ and IgA antibodies against *Apis mellifera* (honeybee), *Polistes* *dominula* (wasp)*,* and *Vespula* *vulgaris* (wasp) venoms.

The basal tryptase level considered was the one measured during the initial assessment.

### Basophil activation test

BAT was performed according to our protocol at Limoges with a custom kit.^16^ Briefly, whole blood cells were primed with IL-3 and incubated at 37°C for 10 minutes. Cell suspensions were mixed with either buffer alone or with positive controls (anti-IgE and anti-IgE receptor antibodies). Venom extracts (also from Jubilant Pharma) were tested at 250, 83, 28, and 9 ng/mL. The mixture was incubated at 37°C for 30 minutes in a water bath. Basophil activation was stopped by adding EDTA buffer. Basophils were labeled using anti-IgE, anti-CCR3, and anti-CD63 antibodies. Erythrocytes were lysed with ammonium chloride buffer for 10 minutes at room temperature. The cells were centrifuged, followed by resuspension in phosphate-buffered saline, then analyzed by flow cytometry (FACSLyric; Becton Dickinson). Basophil response was measured by calculating the area under the curve response. Area under the curve was defined as the integral of the best-fit curve: basophil reactivity (% CD63) = *F*(*x*) μg/mL venom, where *x* stands for allergen concentration, and calculated from 250 ng/mL to 9 ng/mL.

### IgE blocking activity

An aliquot of whole blood (with EDTA) from a venom-allergic individual was washed with phosphate-buffered saline and resuspended in activation buffer. First, 25 μL of the tested serum (from the desensitized patient) was diluted 1:4 and mixed with 75 μL of negative serum pool (serum negative for venom sIgE, taken from a patient with no history of venom allergy). A single target venom concentration (83 ng/mL in RPMI 1640 buffer) was mixed with the tested serum dilution. Second, this venom concentration was also mixed with the negative serum pool (100% control). These 2 dilutions were incubated for 20 minutes at room temperature in parallel with a negative control (RPMI 1640 alone). Then an aliquot of these dilutions was mixed in equal parts with the washed cell pellet and incubated for 30 minutes at 37°C in a water bath. The other steps were performed as described above. Results were expressed as percentage of CD63 and in a percentage calculated versus the 100% control.

### Statistical analysis

Statistical analysis was performed by R v4.2.1 software (www.r-project.org). Qualitative variables were described as numbers and percentages; quantitative variables were described as mean, median, and interquartile ranges (difference between first and third quartile, covering 50% of patients). Comparisons between qualitative variables were carried out by the parametric chi-square, or by the nonparametric Fisher test if necessary. Comparisons between quantitative variables were carried out by the parametric Student test, or by the nonparametric Mann-Whitney test in case of nonnormal data distribution. Correlations were carried out by the parametric Pearson test, or by the nonparametric Spearman test in the case of nonnormal distribution. The evolution of biological variables before and after AIT was compared by Student *t* test for paired series, or by paired Wilcoxon test in the case of nonnormal distribution. Predictive factors for recurrence after stopping AIT were studied by binomial logistic regression. The tests were 2 tailed, and the significance level was set at 5% (*P* < .05).

### Ethical approval

The patients signed an informed consent form approved by the Limoges research ethics committee. The study was registered under the name HyménoLim (87RI22_0062; www.chu-limoges.fr/IMG/pdf/etudes_sur_donnees_registre_public_maj2024_01-3.pdf).

## Results

### Objectives

Our primary objective was to determine if the level of blocking factors at the end of AIT could predict its effectiveness. The outcome measure used was the presence of anaphylactic reaction (grade I to IV according to Ring and Messmer) in case of a new sting after discontinuation of AIT.

The secondary objectives were as follows:

• To describe the characteristics of patients who underwent AIT at the University Hospital of Limoges, including age, sex, occupation, and initial reaction.
• To describe the level of basal tryptase in patients allergic to Hymenoptera treated with AIT.
• To describe the evolution of clinical factors (skin prick test) and biological factors (sIgE, BAT) between initiation and discontinuation of AIT according to type of Hymenoptera.
• To evaluate the correlation between the level of blocking factors and the quantity of sIgG_4_.
• To conduct follow-up of the allergy assessment after discontinuation of AIT.

### Sample description

Since 1985, a total of 387 patients benefited from AIT for Hymenoptera venom at the University Hospital of Limoges. We drew 190 patients for our study from this larger sample. The population showed a majority of AIT for *Vespula* wasp venom, with 141 patients, compared to 44 for bee venom and 5 for *Polistes* wasp venom. As a result of the small sample sizes of the *Polistes* wasp and dual immunotherapy groups, we do not report their results.

Sixty-five percent of the population was male. There were no significant differences in demographics (Table I) or medical history among our patients. Twenty percent of our population was exposed professionally and recreationally, particularly among patients receiving AIT, to bee venom, predominantly beekeepers or their family members. Most patients had initially presented with a grade III reaction according the Ring and Messmer classification. There was no significant difference in severity according to insect (*P* = .478).

**Table I:** Population characteristics

Characteristic	*Vespulavulgaris* (n = 141)	*Apismellifera* (n = 44)	Total (N = 185)	*P* value
Sex				.571
Male	94 (66.7)	27 (61.4)	121 (65.4)	
Female	47 (33.3)	17 (38.6)	64 (34.6)	
Age (years), mean (min, max)	49 (6, 72)	43 (4, 71)	48 (4, 72)	.093
Comorbidity				
Atopy	13 (9.22)	5 (11.36)	18 (9.7)	.912
Asthma	4 (2.84)	1 (2.27)	5 (2.7)	1
COPD	1 (0.71)	1 (2.27)	2 (1.08)	.423
Cardiopathy	6 (4.26)	3 (6.82)	9 (4.86)	.449
Diabetes	4 (2.84)	0	4 (2.16)	.578
Hypertension	20 (14.18)	5 (11.36)	25 (13.51)	.569
AID	9 (6.38)	0	9 (4.86)	.127
Cancer	1 (0.71)	0	1 (0.54)	1
Mastocytosis	4 (2.84)	0	4 (2.16)	.578
Treatment (β-blocker or ACEI)	2 (1.42)	0	2 (1.08)	1

Data are presented as nos. (%) unless otherwise indicated. *ACEI,* Angiotensin-converting enzyme inhibitor; *AID,* autoimmune disease; *COPD,* chronic obstructive pulmonary disease.

### Initial allergy testing

**Fig 1:**
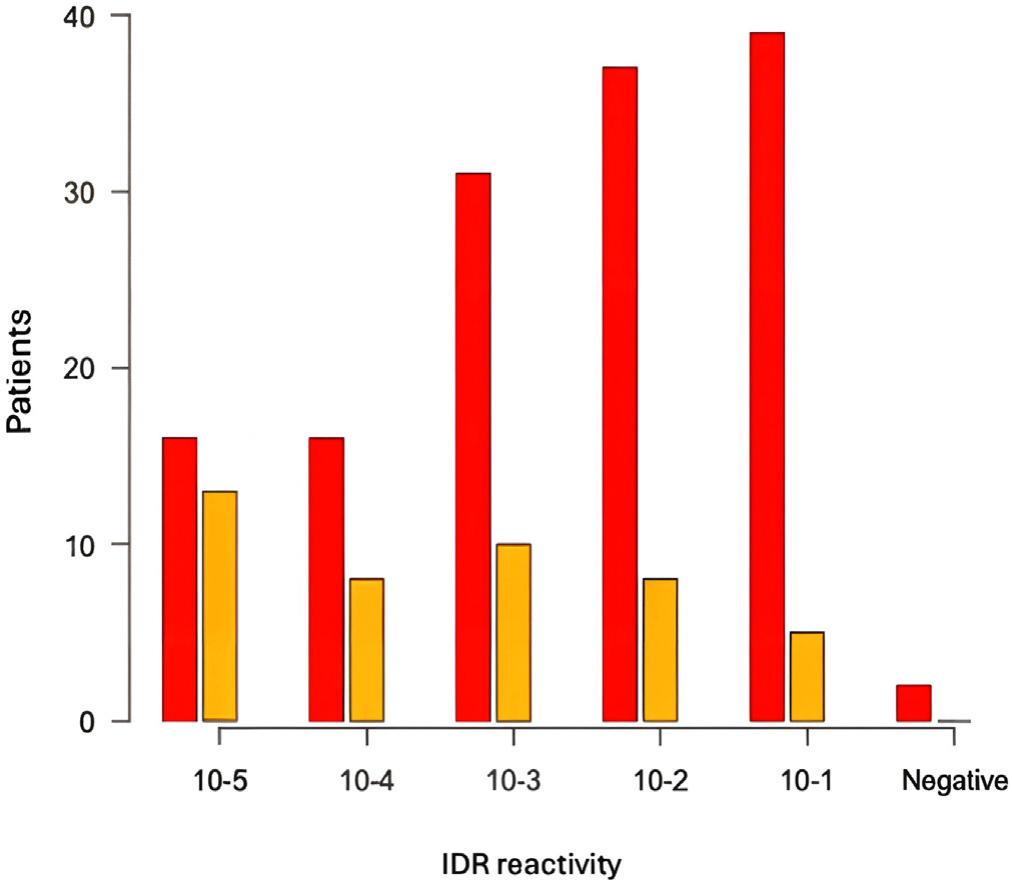
Initial IDT reactivity. *Red, Vespula* wasp; *yellow,* bee.

**Fig 2:**
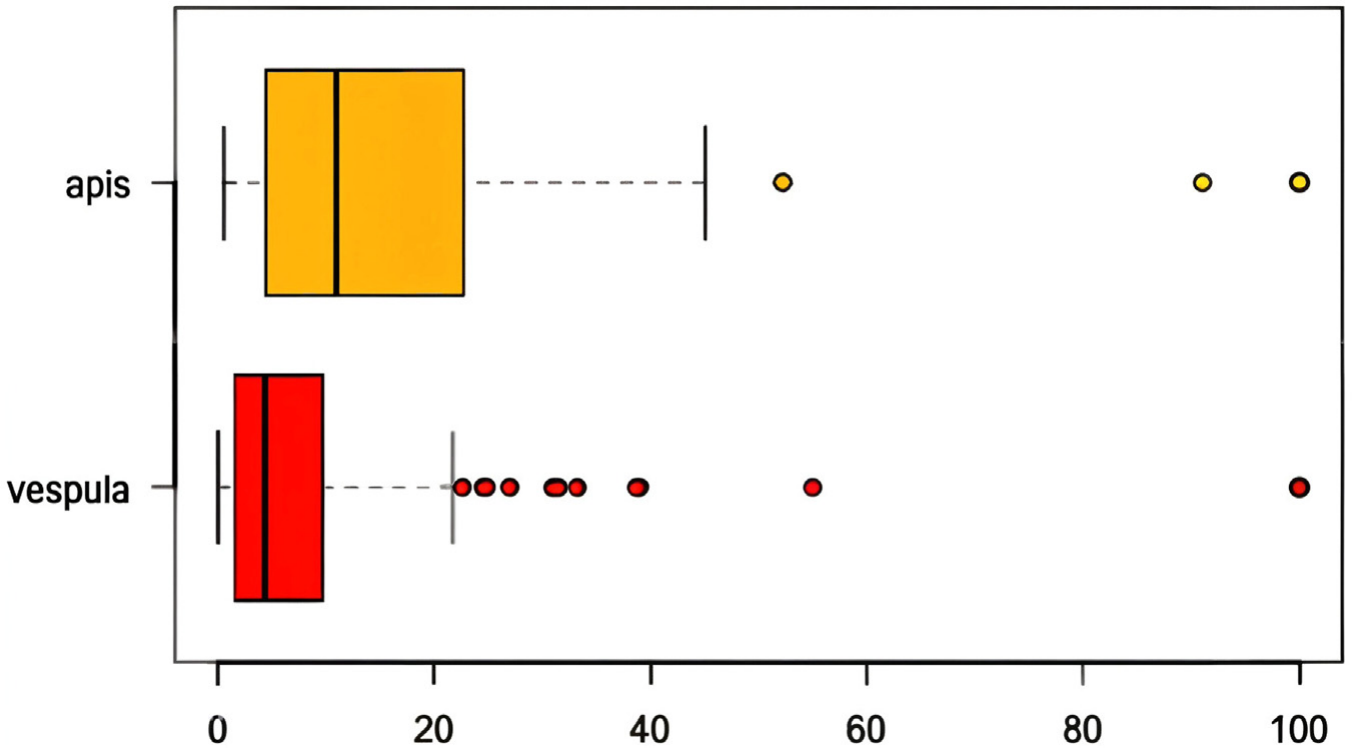
Initial sIgE rates (kU/L). *Red, Vespula* wasp; *yellow,* honeybee.

Regarding the initial allergy assessment of our population, we observed a higher reactivity in the diagnostic tests for bee venom. For example, IDTs were positive at the earliest dilutions (0.001 μg/mL), while those for wasps were mostly positive at the highest dilutions of 10 μg/mL (Fig 1). The sIgE levels were also significantly higher for bee allergy, with a median of 10.9 kU/L compared to 4.4 kU/L for *Vespula* wasp (Fig 2). Most of the BATs showed a strong positive result, but 24% of them were uninterpretable. There was no significant difference in reactivity between insects.

Concerning baseline serum tryptase, the median level was 4.7 μg/L, and there was no significant difference based on venom. Our population included 4 patients with mastocytosis, with an average tryptase level of 44.4 μg/L. Allergen-specific blocking IgG_4_ levels were low at the start of AIT with a median of 0.5 mg/L, but were still better than the values observed in healthy volunteers (0.03 ± 0.04 mg/L). sIgA levels were higher than sIgG_4_, with a median of 1.03 mg/L.

#### *Vespula* AIT

For our largest group of patients who received AIT for *Vespula* wasp venom, there was a notable reduction in their sIgE levels, dropping from an average of 4.4 kU/L to 1.9 kU/L. After immunotherapy, 43% of cases showed negative results in IDTs. BATs also exhibited a significant decrease in reactivity.

Throughout the course of immunotherapy, sIgG_4_ levels showed an increase, although this finding was not statistically significant (*P* = .06). By the end of the treatment, three quarters of the patients displayed positive IgE blocking activity, with a median of 89.4%. Furthermore, there was a correlation observed between IgE blocking activity and sIgG_4_ levels (*P* = .021).

#### Honeybee AIT

**Fig 3:**
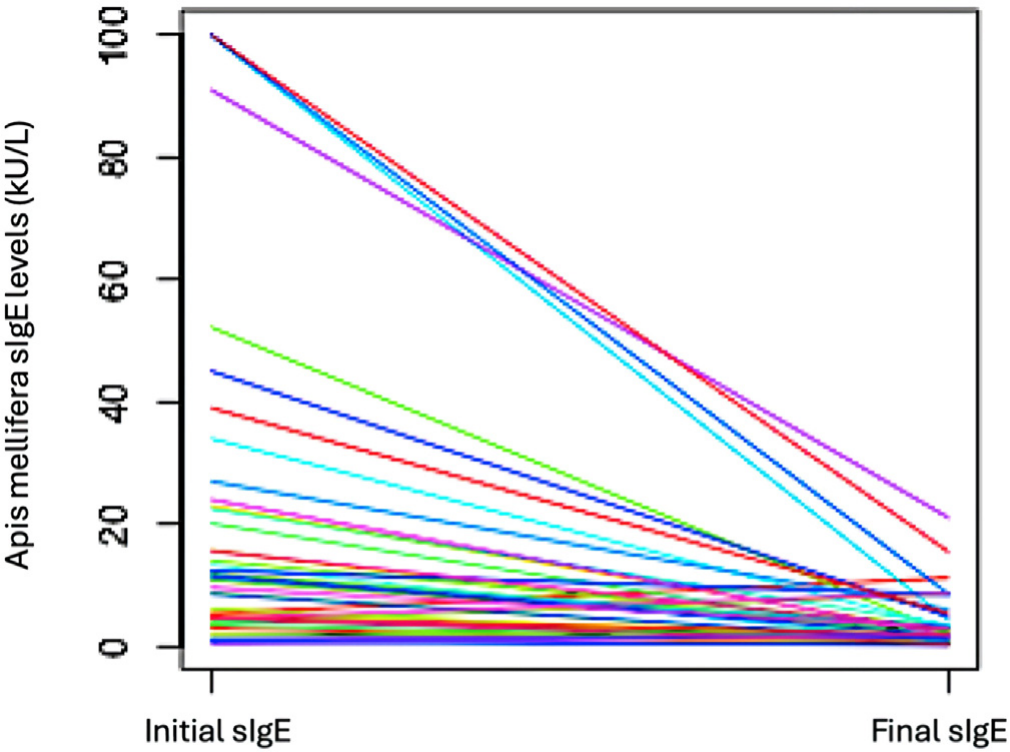
*Apis mellifera* sIgE evolution.

**Fig 4:**
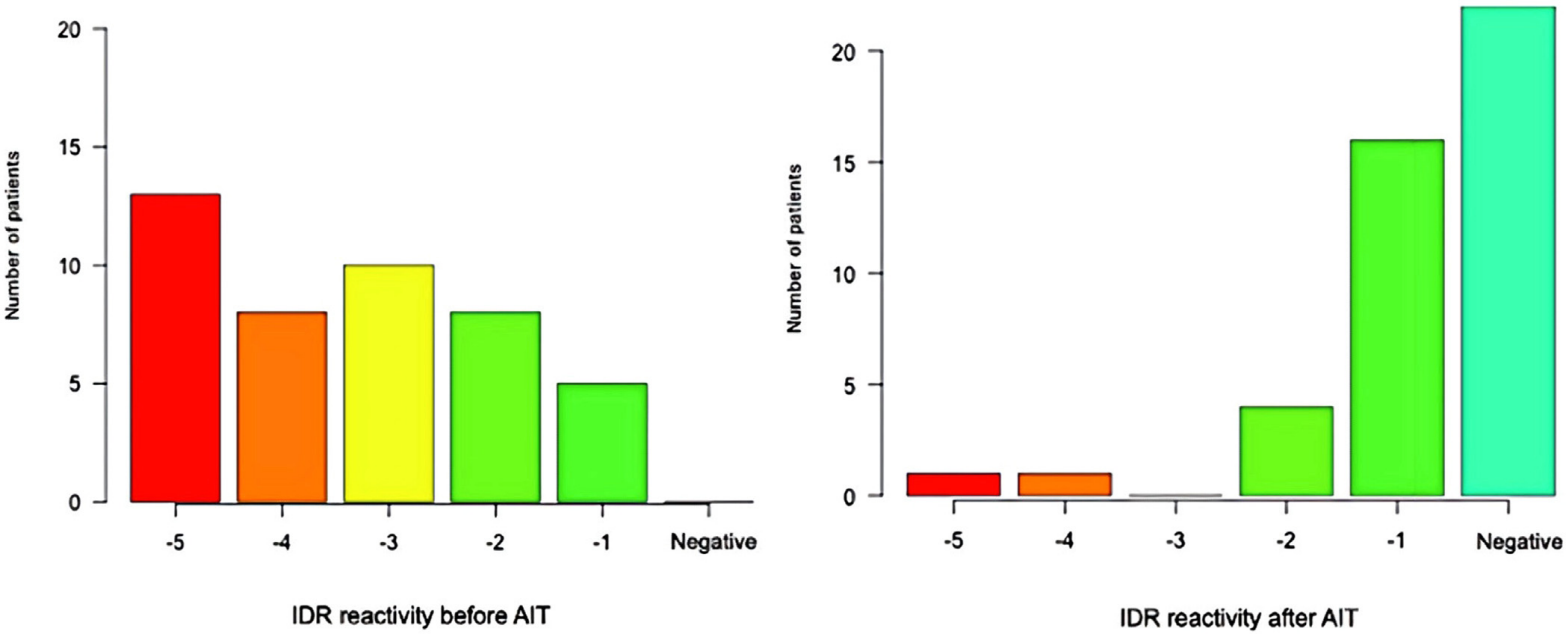
Reactivity evolution of IDTs (*Apismellifera* AIT).

In our study focusing on bee venom, we noticed a consistent decline in the reactivity of diagnostic tests from the start to the end of immunotherapy. Both sIgE levels (Fig 3) and IDTs (Fig 4) exhibited a noticeable decrease. Initially, these tests showed high reactivity, particularly at dilutions of 0.001 μg/mL (10^−5^), but largely turned negative by the end of immunotherapy. At the end of AIT, the median IgE blocking activity stood at 88%, mirroring the findings observed with *Vespula* wasp venom.

### Parameter variation

At the end of AIT, we observed a notable reduction in skin reactivity and sIgE levels. Moreover, there was a significant correlation between the changes in these factors throughout the course of AIT (*P* < .01). During immunotherapy, sIgG_4_ levels showed a significant increase (*P* = .016), whereas sIgA did not exhibit a similar trend. While there was a nonsignificant decrease in basophil activity during AIT, we did not find any correlation between final BAT results and sIgG_4_ levels (*P* = .23) or with IgE blocking activity (*P* = .33).

### IgE blocking activity

**Fig 5:**
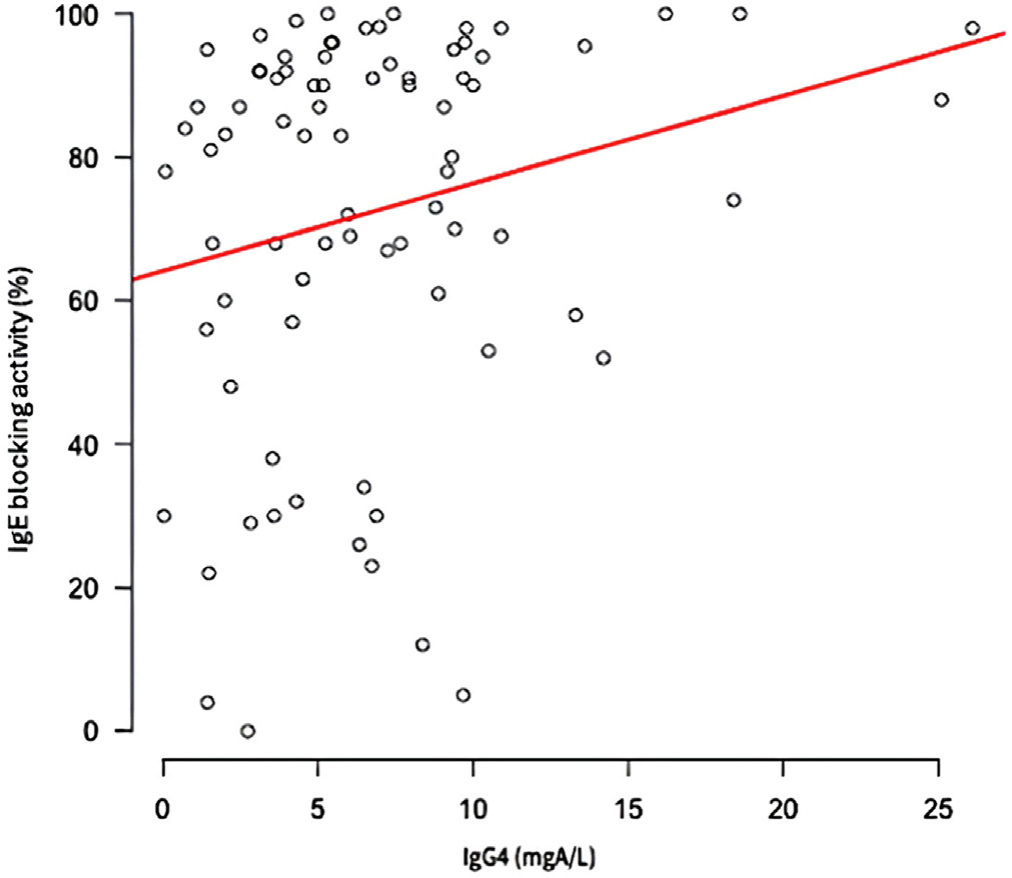
IgE blocking activity/sIgG_4_ correlation.

**Fig 6:**
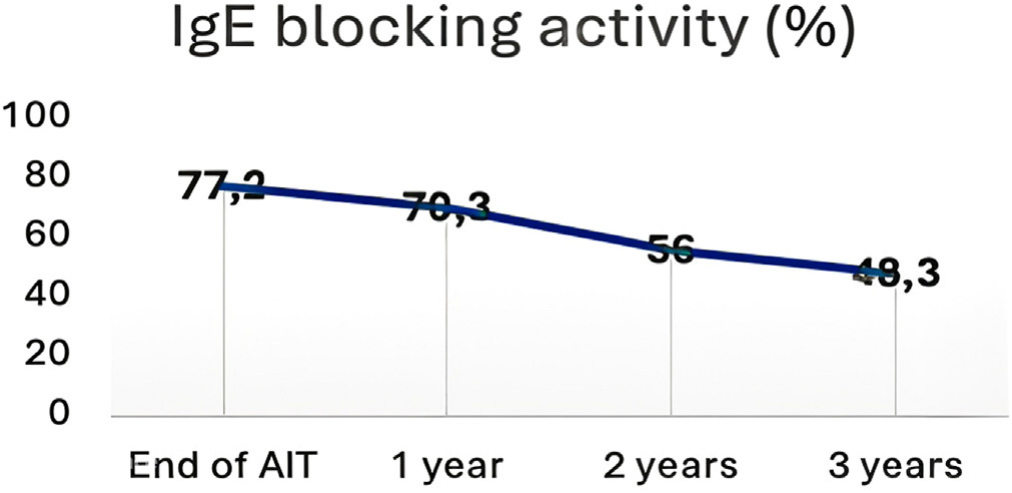
IgE blocking activity after AIT.

Concerning our main objective, the median IgE blocking activity level at the end of immunotherapy was 89%. There was no significant variance in these activity levels by insect venom. We found a significant correlation between IgE blocking activity levels and sIgG_4_ levels (*P* = .021), as illustrated in Fig 5. However, there was no such correlation with sIgA levels (*P* = .66). At the end of AIT, we were able to follow up with approximately 30% of the patients who received complete treatment (5 years), and we found a significant decrease in the rate of IgE blocking activity in the years after venom removal (*P* < .01) (Fig 6).

**Fig 7:**
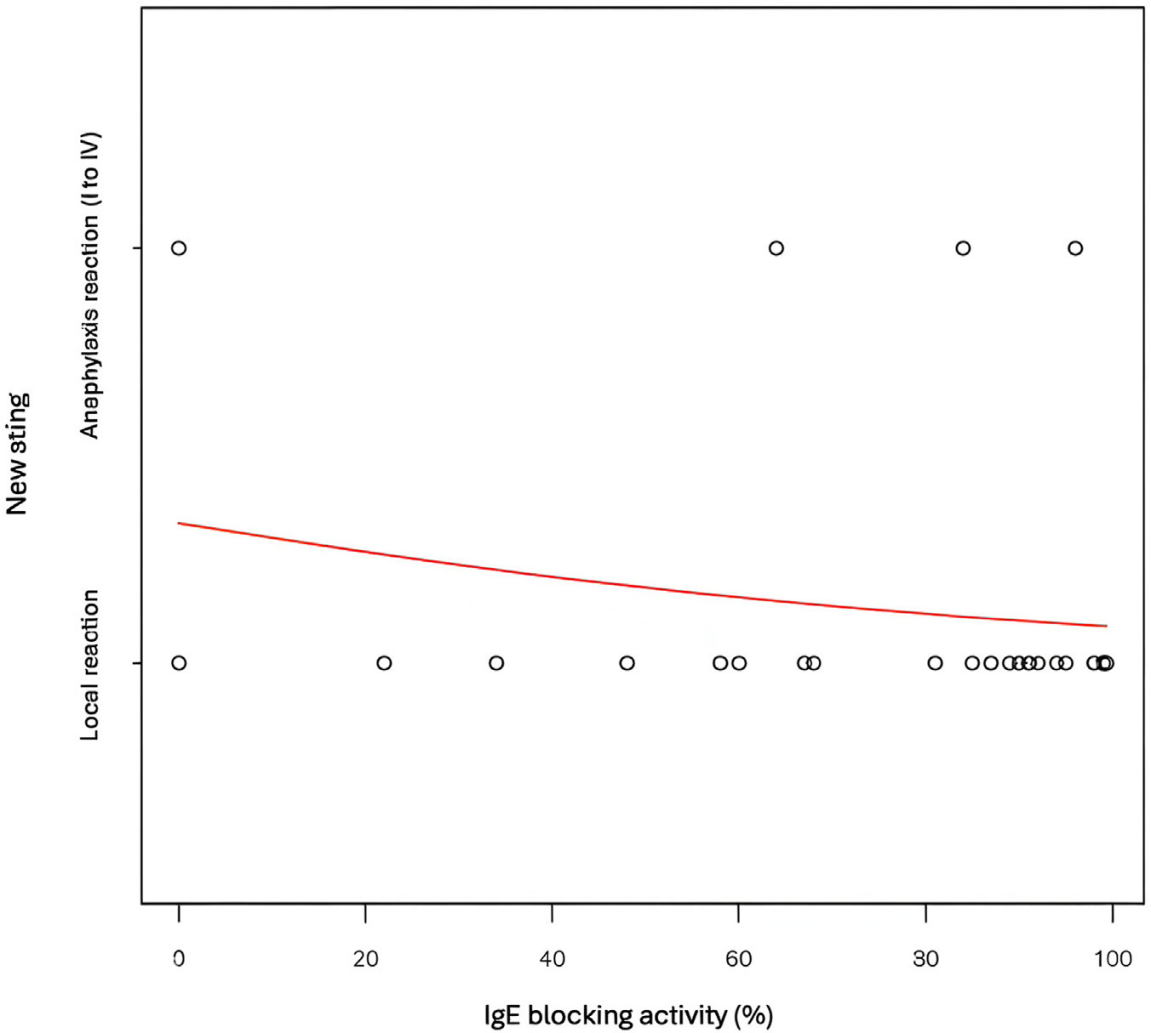
IgE blocking activity and risk of relapse.

Among the 34 patients stung after stopping their AIT, only 4 experienced anaphylactic reactions (11.8%). A binomial logistic regression did not show any interest of IgE blocking activity in predicting the risk of relapse (*P* = .308). Nonetheless, we observed from this regression analysis that there seems to be a tendency for relapse in cases of low IgE blocking activity, as depicted in Fig 7. A receiver operating characteristic curve revealed a sensitivity of 75% and a specificity of 64% when using IgE blocking activity at a threshold of 84.5%.

In addition to IgE blocking activity, other parameters were evaluated to search for a predictive marker of relapse. The history of atopy showed significant relevance in predicting relapse (*P* = .006). An optimal efficacy of AIT was observed at 88.2%, with a relapse rate of 11.8%. However, these data highlight the need for patients to maintain an emergency kit for life.

## Discussion

The Limousin, where we conducted the study, is a rural region with high exposure to Hymenoptera. This study revealed significant occupational and leisure exposure.

The strength of our study is that it is conducted over a long period of time, with a large cohort of allergen-desensitized patients. The findings are consistent with current data. However, the retrospective nature of the study introduces biases such as recall bias, with several data missing. Sample sizes were different, with a majority of *Vespula* venom cases. This can be explained by the need for lifelong AIT in exposed patients, as is the case with bee venom for beekeepers. Therefore, few beekeeper patients were included because we focused on completed AITs. Last, there is a lack of statistical power regarding patients who experienced relapse after AIT (n = 4). This is a statistical limitation, but from a clinical perspective, it demonstrates the effectiveness of our treatment: AIT is well tolerated, with a good efficacy of 88.2%, consistent with current studies. Among the 185 patients who benefited for the treatment, only one patient stopped therapy early as a result of adverse effects. Despite the low relapse rate, patients should still carry an emergency kit after AIT discontinuation.

Regarding our primary goal, we did not demonstrate significant usefulness of IgE blocking activity in predicting potential relapse; we showed a tendency to relapse in case of low IgE blocking activity. More studies with higher statistical power are necessary. However, our study has enabled us to examine a new threshold of 84.5%. IgE blocking activity is well correlated with sIgG_4_ levels, but not with sIgA. It is easy to assume that the route used for venom administration favors the production of IgG rather than IgA. The latter is mainly produced via the sublingual route.^17^ The potential involvement of IgA cannot be excluded, as depletion studies have not been conducted. Studies have already provided evidence for the functional role of IgG_4_ and even IgG_1_.^12^ Moreover, the significant increase in sIgG_4_ can be useful for monitoring treatment response during AIT through regular measurements.

IgE blocking activity is not commonly used in routine practice, partly as a result of the use of blood from an allergic subject donor, which needs to be changed regularly. Further studies are necessary to standardize IgE blocking activity. We demonstrated a significant decrease in the response of BATs, IDTs, and sIgE at the end of immunotherapy. Concerning BATs, 24% of them were uninterpretable as a result of nonresponse to therapy due to drug interference or variability in FcεRI expression. The initial test results for bee venom show higher reactivity than for wasp venom; for example, median sIgE for bee was 10.9 kU/L compared to 4.4 kU/L for *Vespula* wasp. When considering AIT for certain patients, a lower test reactivity for *Vespula* venom should not be underestimated.

We had few cases of double AIT, possibly because inhibition tests and recombinant assays were less commonly performed in previous years. Furthermore, the inhibition test, despite its lack of standardization, is commonly conducted in Limoges, but not in all centers. Our patients with mastocytosis were not representative of the incidence of this condition in the general population. Similar to bee venom patients, this is likely because AIT is lifelong in this population, and these patients were therefore not included in our study.

### Conclusion

Allergy to Hymenoptera venom is a severe and potentially life-threatening one that greatly affects quality of life. The only effective long-term treatment is AIT, which reduces the risk of anaphylaxis. Antibody and cellular responses allow us to understand some of the mechanisms of AIT but cannot be applied as biomarkers. However, sIgG_4_, in correlation with IgE blocking activity, could be useful for monitoring treatment response. Additional studies are necessary to gain a thorough understanding of the composition of inhibitory antibodies and establish standardized protocols for this assay to decide whether to cease AIT.

## Disclosure statement

Disclosure of potential conflict of interest: The authors declare that they have no relevant conflicts of interest.

**Clinical implication**

IgE blocking activity is correlated with the amount of sIgG_4_, and low levels of activity may suggest a likelihood of relapse.
