# Supplementary material for: Monitoring and stopping Hymenoptera venom immunotherapy: Contribution of IgE blocking activity
Source: J Allergy Clin Immunol Glob. 2024 Aug 24;3(4):100329. doi: 10.1016/j.jacig.2024.100329 (PMC11415340; doi:10.1016/j.jacig.2024.100329)
Supplement: Supplementary data [file mmc1.docx]

**Supplementary data**

Evolution of Vespula AIT parameters

| Vespula AIT (n = 141) | | | |
| --- | --- | --- | --- |
| **Test** | **Initial** | **Final** | ***p - value*** |
| **IDR** | | | ***< 0.01*** |
| **10^-5^ (%)** | 16 (11.35%) | 0 (0.00%) |  |
| **10^-4^ (%)** | 16 (11.35%) | 1 (0.71%) |  |
| **10^-3^ (%)** | 31 (21.99%) | 7 (4.96%) |  |
| **10^-2^ (%)** | 37 (26.24%) | 29 (20.57%) |  |
| **10^-1^ (%)** | 39 (27.66%) | 42 (30.50%) |  |
| **Negative (%)** | 2 (1.42%) | 61 (43.26%) |  |
| **sIgE (kU/L) (median)** | 4.4 | 1.9 | ***< 0.01*** |
| **BAT (AUC) (median) (Q25 ; Q75)** | 6700 (298 ; 15242) | 1964 (279 ; 6304) | ***< 0.01*** |
| **sIgG4 (mg/L) (median)** | 0.5 | 5.49 | *0.06* |

Evolution of Honeybee AIT parameters

| Honeybee AIT (n = 44) | | | |
| --- | --- | --- | --- |
| **Test** | **Initial** | **Final** | ***p-value*** |
| **IDR** | | | ***< 0.01*** |
| **10^-5^ (%)** | 13 (29.55%) | 1 (2.27%) |  |
| **10^-4^ (%)** | 8 (18.18%) | 1 (2.27%) |  |
| **10^-3^ (%)** | 10 (22.73%) | 0 (0.00%) |  |
| **10^-2^ (%)** | 8 (18.18%) | 4 (9.09%) |  |
| **10^-1^ (%)** | 5 (11.36%) | 16 (36.36%) |  |
| **Negative (%)** | 0 (0.00%) | 22 (50%) |  |
| **sIgE (kU/L) (median)** | 10.9 | 2.11 | ***< 0.01*** |
| **BAT (AUC) (median) (Q25 ; Q75)** | 9720 (327 ; 17800) | 1814 (336 ; 9783) | *0.22* |
